# 290. Prediction of Severe Clostridioides difficile Infection Using Isolate Genomic Variation Influenced by Patient Comorbidity Score

**DOI:** 10.1093/ofid/ofad500.362

**Published:** 2023-11-27

**Authors:** Emily C Maggioncalda, Evan Snitkin, Vincent B Young, Krishna Rao

**Affiliations:** University of Michigan Medical School, Ann Arbor, Michigan; University of Michigan, Ann Arbor, MI; University of Michigan, Ann Arbor, MI; Department of Internal Medicine, Infectious Diseases Division University of Michigan, Ann Arbor, Michigan, Ann Arbor, MI

## Abstract

**Background:**

*Clostridioides difficile* infection (CDI) is a complex disease involving host and microbial factors. The influence of patient clinical state complicates identification of pathogen genetic variation associated with disease progression. Stratification of patients by underlying comorbidity may improve prediction of important *C. difficile* genetic variants by controlling for confounding.

**Figure:**
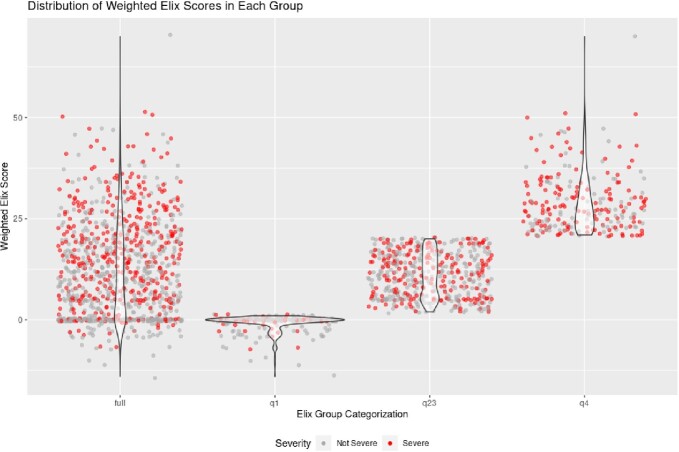

Distribution of the weighted Elixhauser scores for patients across the different sub-analyses. Q1: "healthiest" patients, Q23: "intermediate" patients, Q4: "sickest" patients.

Descriptive Statistics of Patient Data Sets

**Figure:**
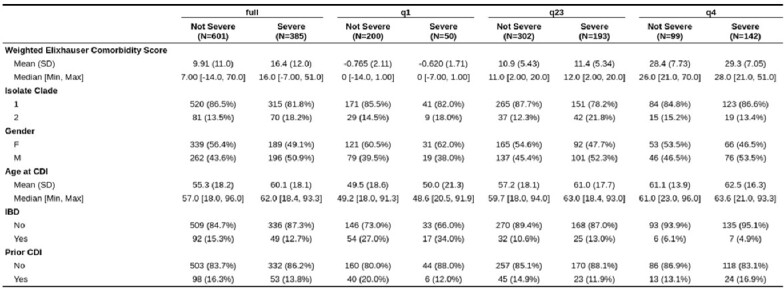

Table outlining the patient descriptive statistics of the full dataset as well as the sub-analyses datasets.

**Methods:**

Analysis consisted of 986 patient/*C. difficile* pairs. Severe patient outcome is a white blood cell count of > 15,000 per mL or a creatinine level of >1.5 mg/dL. Weighted Elixhauser score is a comorbidity measure utilizing patient ICD-10 codes, returning a standardized measure of patient morbidity, weighted by hospital mortality risk. Weighted Elixhauser was used to split the full dataset into sub-analyses based on underlying patient comorbidity: 1st quartile (q1, "healthiest"), 4th quartile (q4, "sickest"), 2nd & 3rd quartile (q23, "intermediate"). Complete genomic variation of the infecting *C. difficile* strains, as well as sets curated by biological annotation (“biological”) or genome wide association study (“handsoff”) were used as the features of interest. Logistic regression was performed using the R package mikropml.

Overview of the Machine Learning Workflow

**Figure:**
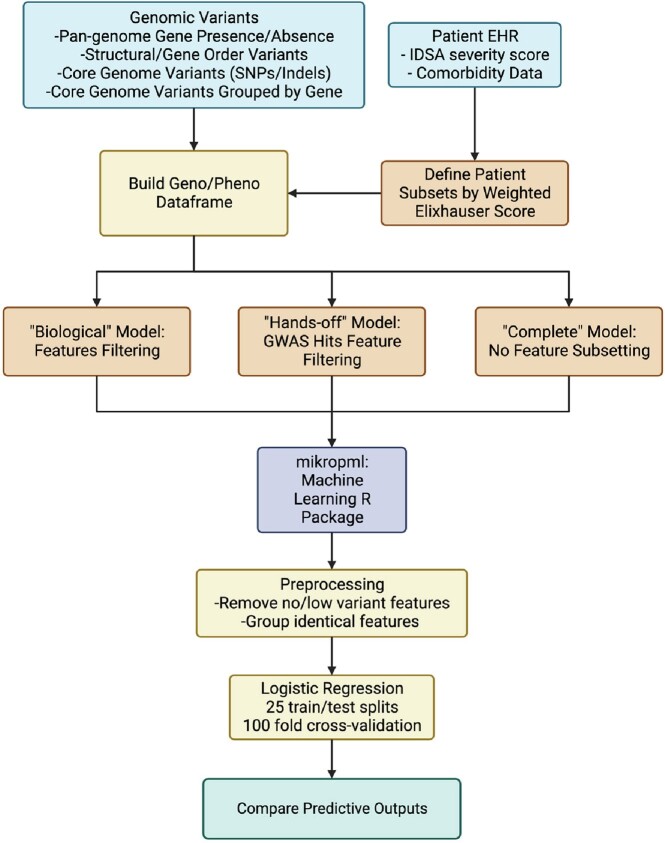

Genetic variant features for the C. difficile isolates were derived from the Panaroo presence/absence matrix and the structural variant matrix, as well as core genome single nucleotide polymorphisms (SNPs) and insertion/deletions (indels), both individually and grouped by locus ID. These genetic features were then combined with the different phenotype splits defined by weighted Elixhauser comorbidity score. The feature set was then either kept as is, or was filtered further using biological annotation information derived from eggNOG and KEGG text descriptions, ortholog families, and COG categories, or by "Hands-off" annotation using only features that were returned as hits from one of two phylogeny based Genome Wide Association Study (GWAS) algorithms, treewas or hogwash. These feature sets and outcome measures were then fed into mikropml, and 25 separate train/test splits of the data were performed, each with 100-fold cross-validation, to come to final predictive models for severity outcome.

Feature Counts for the different Feature Sets and Phenotype Splits

**Figure:**
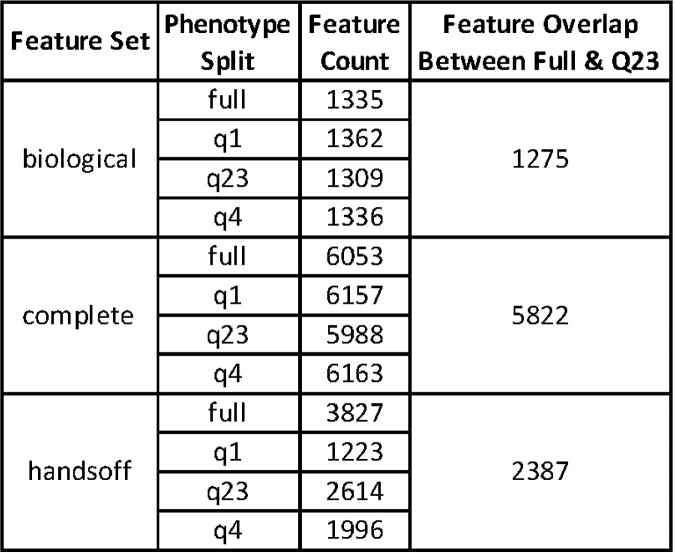

The features included for each analysis was dependent on 1) the strains present in the phenotype split and 2) the filtering method for the feature set. Since not all genetic variants were present across all strains, the subset of strains included as part of each phenotype split can lead to different genetic features being included. If the feature set was being filtered for biological importance then the features were required to include annotations relevant to established C. difficile pathogenesis mechanisms. If the feature set was being filtered by Genome Wide Association Study (GWAS) significance the feature would only be retained if it was marked as significant by at least one of two phylogeny based GWAS methods, hogwash or treewas. The overlap in the feature sets between the full phenotype split and the Q23 split is also indicated.

**Results:**

The greatest improvement in Area Under the Curve (AUC) was observed between the full patient data and q23 split using complete genomic variation (full = 0.532 , q23 = 0.596). AUC was significantly impacted by patient phenotype split (p value = < 2e-16) but not by genomic feature sets (p value = 0.83). Improvement in AUC primarily derived from removal of the “healthiest” patient split, as the full patient data and q1 split performed similarly (full-q1 p value = 0.27), while improvement in AUC was observed for the “intermediate” and “sickest” patient splits (full-q23 p value < 1e-7, full-q4 p value < 1e-7).

**Figure:**
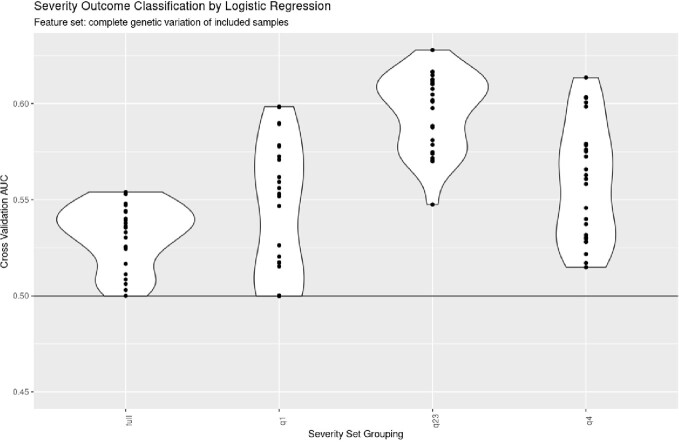

Comparison of logistic regression performance for the complete feature set across the different phenotype splits. There is a significant improvement in area under the curve (AUC) when the outlier "healthiest" and "sickest" patients are removed and only the central quartiles remain (q23). The outer quartiles, q1 and q4, have large variations in their AUCs depending on the train/test split of the data used for model generation.

**Figure:**
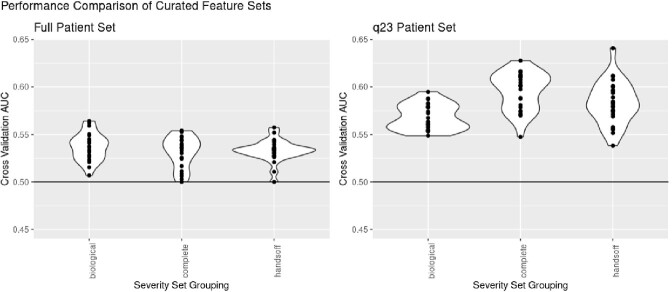

The use of curated feature sets did not demonstrate any statistically significant improvement over the complete genetic variation feature set. Only the full and q23 phenotype split results are shown. The "handsoff" model using GWAS filtered hits demonstrated a trend towards less variation than the other models in the full phenotype split, but this was not statistically significant. The biological annotation filtered set trending towards performing slightly better in the full phenotype split, but this trend did not carry to the q23 phenotype split.

Area Under the Curve (AUC) Model Performance Summaries

**Figure:**
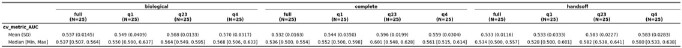

AUC performance summaries for all combinations of phenotype splits and genomic feature sets. cv_metric_AUC = cross validation AUC.

**Conclusion:**

Reduced model performance when including the “healthiest” patients could indicate a greater influence of host factors versus infecting *C. difficile* strain genetics. Integration of patient microbiome or serum cytokine data may improve predictive capacity for these patients. Exploration of predictive features for the q23 and q4 models may derive novel insights into CDI pathogenesis.

**Disclosures:**

**Vincent B. Young, MD, PhD**, ASM: Senior Editor for mSphere Journal|Debiopharm: Consultant|mSphere: Senior Editor|Vendanta Biosciences: Consultant **Krishna Rao, MD, MS**, Merck & Co.: Grant/Research Support|Rebiotix: Advisor/Consultant|Seres Therapeutics: Advisor/Consultant|Summit Therapeutics: Advisor/Consultant

